# EEG dynamics and neural generators of psychological flow during one tightrope performance

**DOI:** 10.1038/s41598-020-69448-3

**Published:** 2020-07-24

**Authors:** A. Leroy, G. Cheron

**Affiliations:** 1 Laboratory of Neurophysiology and Movement Biomechanics, Université Libre de Bruxelles, Brussels, Belgium; 2 Laboratory of Electrophysiology, Université de Mons, Mons, Belgium; 3 Haute Ecole Provinciale du Hainaut-Condorcet, Mons, Belgium

**Keywords:** Neuroscience, Cognitive neuroscience, Attention

## Abstract

Psychological “flow” emerges from a goal requiring action, and a match between skills and challenge. Using high-density electroencephalographic (EEG) recording, we quantified the neural generators characterizing psychological “flow” compared to a mindful “stress” state during a professional tightrope performance. Applying swLORETA based on self-reported mental states revealed the right superior temporal gyrus (BA38), right globus pallidus, and putamen as generators of delta, alpha, and beta oscillations, respectively, when comparing “flow” versus “stress”. Comparison of “stress” versus “flow” identified the middle temporal gyrus (BA39) as the delta generator, and the medial frontal gyrus (BA10) as the alpha and beta generator. These results support that “flow” emergence required transient hypo-frontality. Applying swLORETA on the motor command represented by the tibialis anterior EMG burst identified the ipsilateral cerebellum and contralateral sensorimotor cortex in association with on-line control exerted during both “flow” and “stress”, while the basal ganglia was identified only during “flow”.

## Introduction

Over 40 years ago, Csikszentmihalyi^1^ first introduced the concept of psychological “flow”, defined as a singular mental state accompanying exceptional performance, which was later popularized as being “in the zone”^2^. It is generally accepted that this exceptional state emerges from a clear goal that requires action, and a perfect match between specific skills and challenges^3–5^. It is a unique sensation, accompanied by a transformation of time, which most commonly occurs in persons engaged in high-skill motor practices, such as champion athletes^6,7^ and musicians^8–11^.

Psychological “flow” may also be considered a specific state of consciousness requiring involvement of the cortical areas participating in the neural correlates of consciousness (NCC). The NCC was initially defined by Crick and Koch^12^ in 1990 as *the minimal neuronal mechanisms jointly sufficient for any specific conscious experience,* and the full NCC is considered the union of all content-specific NCCs^13,14^. We hypothesized that flow identity may be an emergent conscious experience involving specific recruitment among the neural network of the NCC. This notion is supported by the idea that some brain functions necessitate an efficient network of connections and once such coordinated complexity is attained, the emergent properties of consciousness happen, producing the flow sensation. As this sensation goes along with or follows movement, a functional tradeoff between external and internal force must be continuously controlled. The sensation of flow would emerge in a particular physiological state where (1) an appropriate central resting state, including memorized items and motivation is present, (2) the initial intention must be translated by the descending motor commands to the muscles in order to generate forces and displacements, and (3) the ascending somesthetic signals must produce ideal feedback sensations closing the loop between action and sensation^3^.

We further aimed to investigate the complex dialogue between the explicit system of the frontal and medio-temporal lobe involved in off-line cognition, including on-line top–down regulation of attention, and the implicit system, fast and intuitive based on the cortico-basal ganglia loop, promoting skill-based knowledge efficacy^3,4^. Based on these seemingly antagonistic functions, we set out to experimentally test whether the flow state might emerge from a transient hypofrontality^15,16^ that renders the implicit system free from explicit interference.

Motivated by growing interest in the uniqueness of the individual human brain, we recently undertook an experimental search for the emergence of a singular brain state corresponding to psychological “flow”. Despite the presence of diverse artefacts related to the execution of whole-body movements^17^, the use of electroencephalography (EEG) recordings represents a new field of interest^18–20^. Additionally, recent advances in the high-density EEG approach, coupled with inverse modelling methods^21–25^ for the detection of neural cortical and subcortical generators^26^, have paved the way for electrophysiological exploration of the flow state experienced in human participants.

There are many outstanding questions in this field. What must happen in the brain for a person to experience “flow”? Does flow emergence require the recruitment of certain subcortical regions and/or the activation of specific “flow” cortical areas? We thus predict that different EEG generators should be specifically present during this mental state. Among various sports, tightrope activity is particularly attractive for examinations of “flow” because of the highly restrictive field of action, and the requirement for constant exertion of optimal balance control. Thus, in the present study, we examined the brain of a tightrope performer during a performance, to analyze the brain dynamics while crossing a cable at an altitude of 15 m.

Electroencephalography (EEG) recording on the surface of the human scalp during motor and cognitive behavior in ecological situation offers the possibility of scientifically exploring these basic questions in humans. EEG signals are considered to result from the synchronous neuronal activity of local field potentials distributed into temporally and spatially coordinated networks of neurons. EEG signals represent more than the oscillation of the membrane potentials of the neurons; they also represent the activity of the glial cells that contribute to the generation of beta-gamma^27^ and slow-wave activity^28,29^. The reactivity of EEG signals has been mainly studied in response to sensory input or during cognitive tasks, and less commonly during motor behavior. Since EEG signals represent the dynamics of the brain state, it is important to quantify the part of the activity in the EEG signal that is devoted to its downstream impact on motor behavior.

Investigation of the individual brain is often mentioned by the neuroscience community^30^ as one of the main challenges for the future of this field. We believe that such investigations should include EEG recordings to explore the electrical brain dynamics during a single performance in an individual brain. Tightrope performance represents an ideal behavioral situation, in which the subject’s attention is focused on their body equilibrium during perfect locomotion on a straight cable. Here we report the first analysis of the brain of an professional tightrope performer using high-density E,EG, coupled to electro-oculography (EOG), electrocardiography (EKG), and electromyography (EMG) recordings, before, during, and after walking on a long cable (160 m) at an altitude of 15 m under ecological conditions.

We hypothesized that in spite of the fact that flow sensation rarely occurs, if this tightrope performance was perfectly realized by combining high skill and real pleasure sensation, it would involve specific EEG dynamics supported by the participation of neural generators corresponding to the experienced flow. Our findings verified this hypothesis. Moreover, by chance, the performer encountered an unexpected challenge during the recorded performance. This unexpected event enabled us to divide the tightrope crossing into four different periods, and to determine that the participant-reported flow sensation occurred only during the first part of the cable crossing, and not after the unexpected event.

## Results

### Description of the tightrope performance and critical periods

**Figure 1:**
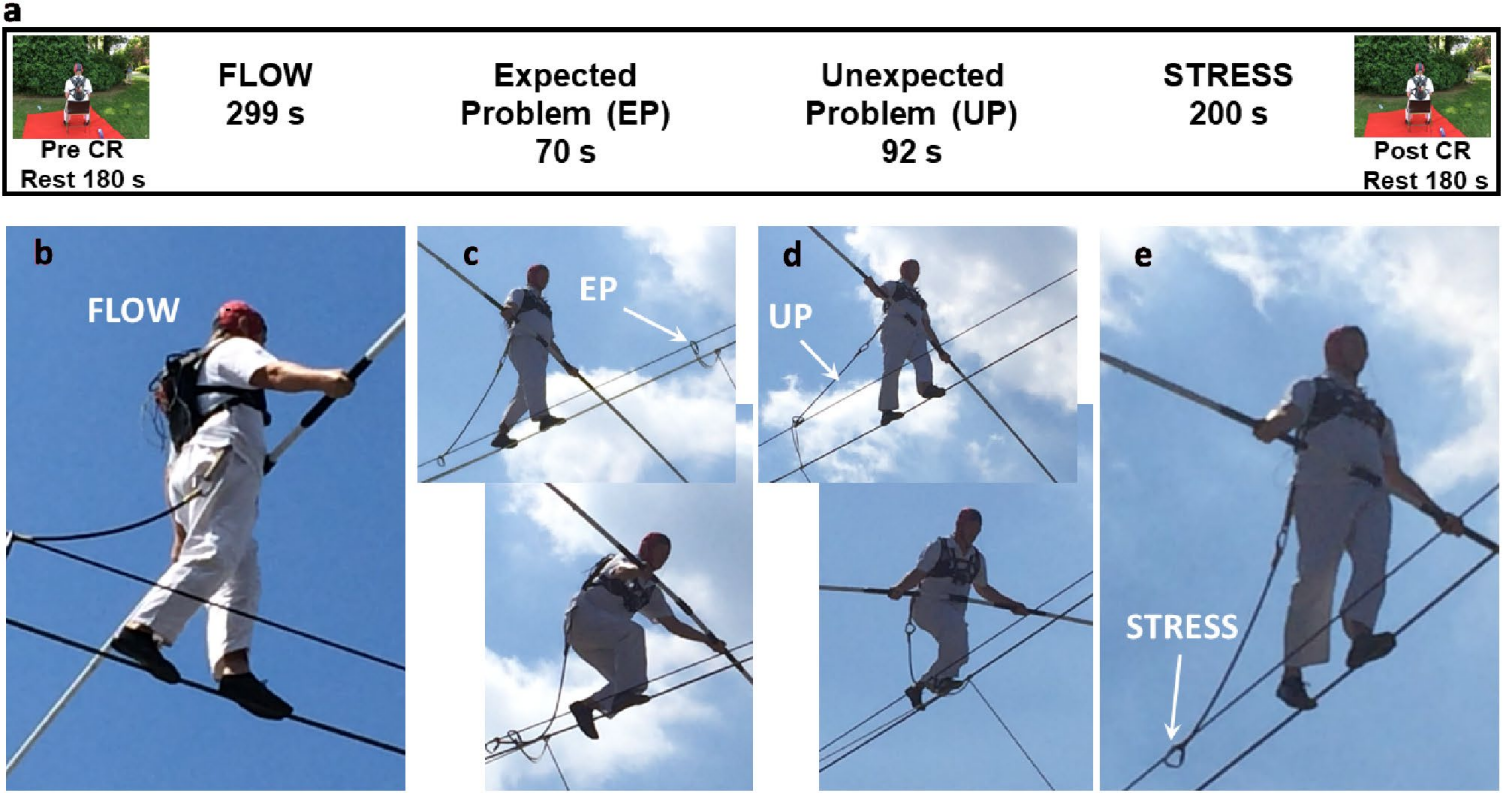
Tightrope performance under outdoor conditions. **a** Timeline of the experimental settings, including pre-crossing (Pre-CR) and post-crossing (Post-CR) rest periods. The tightrope walker was equipped with an EEG cap, comprising EKG, EOG, and EMG electrodes, and walked across a wire under normal performance conditions at a height of 15 m. Four different periods were defined during the crossing (CR): **b** quiet period (FLOW), **c** expected problem period (EP), **d** unexpected problem period (UP), and **e** stress period (STRESS). White arrows indicate the change of the carabiner (**c**), the unexpected carabiner blockage (**d**), and the concern regarding this problem (**e**).

The entire recording session lasted 56.4 min, and the total duration of cable crossing was 13.24 min (Fig. 1). The tightrope walking session was preceded by a 180-s mental preparation period (Pre-Crossing), during which the subject had his eyes closed and was focused on his future action. When crossing a long cable (160 m in the present event), the performer must stop mid-way to pass the lifeline to the other side of the fixing cable, which helps to stabilize the long crossing cable. This action requires that the tightrope walker sit on the cable, unhook the lifeline, and move the lifeline to the other side of the fixing cable. This expected action was perfectly realized, the lifeline was replaced, and the crossing was re-initiated.

**Figure 2:**
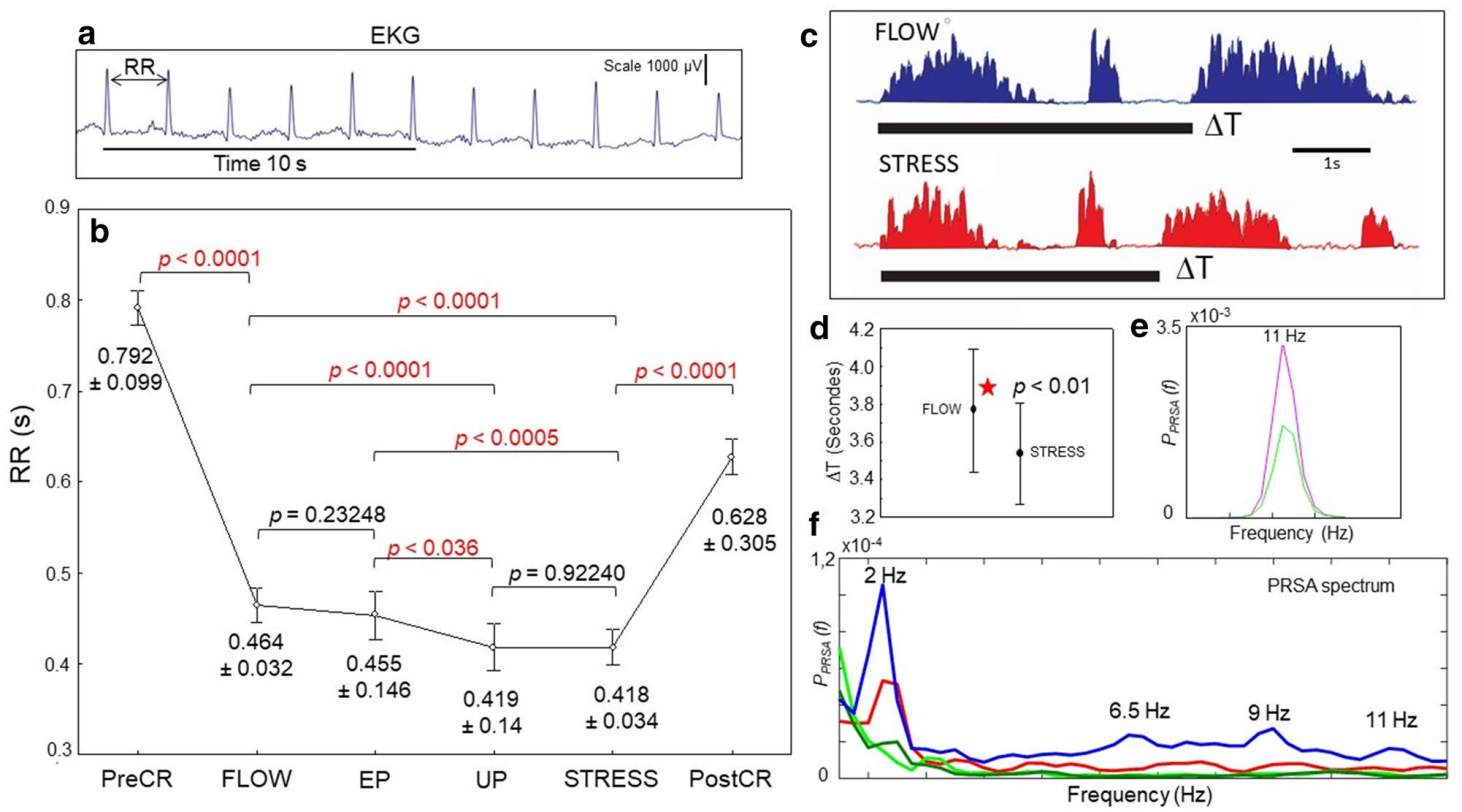
Main physiological characteristics before (PreCR), during (FLOW, EP, UP, and STRESS), and after (PostCR) the tightrope crossing performance. **a** EKG recording during the crossing, with measurement of the heartrate (RR) after identification of the R peak of the QRS signals. **b** RR evolution and statistical comparison (one-way ANOVA) before crossing, during the four periods of crossing, and after crossing. **c** Rectified EMG recordings from the left tibial anterior (TA) muscle during the FLOW (blue) and during the STRESS (red). Black lines show the time between two successive long bursts (∆t). **d** Statistical analysis of the time between two successive long burst of the left TA muscle (∆t) during FLOW crossing and STRESS crossing. **e** Phase-rectified signal averaging (PRSA) between 0 and 20 Hz performed on FCz during rest, showing the dominant alpha peak at 11 Hz (Pre-CR in green; Post-CR in pink). **f** PRSA performed on FCz signals during the four crossing conditions—FLOW (Blue), EP (Green), UP (Green light), and STRESS (Red)—between 0 and 12 Hz, showing few major peaks of delta (~ 2 Hz), theta (~ 6.5 Hz), and alpha (9 and 11 Hz) rhythm.

In the recorded performance, the tightrope walker took a few steps forward after moving the lifeline, and experienced a sudden block of the snap hook. This unexpected event required the performer to take a few steps back to fix his carabiner. These two interruptions of the tightrope crossing (one expected and the other unexpected) allowed us to split the cable crossing performance into four parts: (1) a quiet period (FLOW) of 6.43 min before the first sitting; (2) the expected problem period (EP) of 1.09 min; (3) the unexpected problem period (UP) of 2.84 min; and (4) a stress period (STRESS) of 2.88 min (Fig. 1a–e). Upon completion of the crossing, and after descending the ladder and answering the journalist’s questions, the performer ended the recording session with a 3-min relaxing period with his eyes closed (Post-Crossing). In accordance to the significant heartrate (RR) difference between the FLOW and the STRESS periods (Fig. 2a,b), the Flow Short Scale questionnaire confirmed that these periods were psychometrically different (Supplementary information).

### EMG pattern analysis

The manner of walking on the cable—represented here by the EMG pattern of the tibialis anterior (TA) muscle—differed between the first and third periods of the crossing. The EMG pattern of the TA muscle showed long and short burst sequences, corresponding to a glissade movement of the foot along the cable, followed by a rapid elevation of the foot, respectively (Fig. 2c). The duration of the long burst was 1.5 ± 0.4 s during the FLOW, and 1.3 ± 0.3 s during the STRESS (*P* < 0.05). The time between two successive long bursts producing glissade movement of the same foot (∆t) was 3.81 ± 0.36 s during the FLOW, and 3.54 ± 0.27 s during the STRESS (*P* < 0.01) (Fig. 2d). This global time shortening observed during the STRESS with respect to the FLOW was considered an indication of the mental preoccupation due to the previous occurrence of the unexpected problem.

### FFT analysis with phase-rectified signal averaging (PRSA)

We recorded a classical alpha peak (11 Hz) during the rest state, in both the pre-and post-crossing periods (Fig. 2e). This revealed that alpha oscillation was the dominant rhythm during the pre-crossing and post-crossing rest conditions (eyes-closed meditation state), with no peak frequency shift (Fig. 2e) between these two periods, which are considered particularly crucial for the performer. PRSA analysis demonstrated a delta rhythm peak at about 2 Hz on FCz during the tightrope performance (Fig. 2f), mainly during the FLOW. Additionally, a clear alpha peak was present during the FLOW (Fig. 2f), which disappeared during the EP, UP, and STRESS. This may indicate that the FLOW period, which the participant reported as being close to the ‘flow’ state, was accompanied by alpha oscillations.

### EEG sources during pre-crossing and post-crossing periods

**Figure 3:**
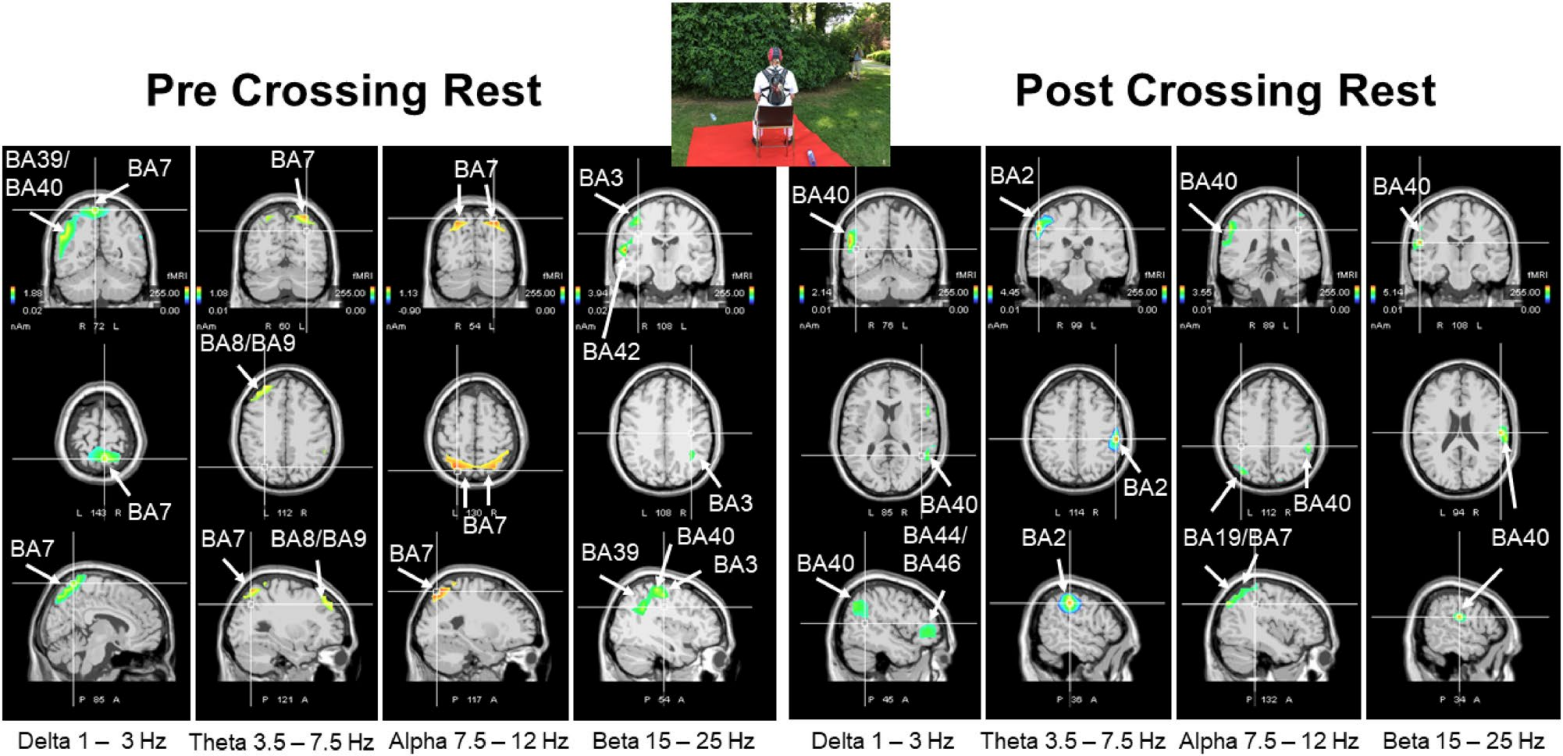
Results of swLORETA during the pre-crossing (left) and post-crossing (right) rest periods for delta, theta, alpha, and beta oscillations. Analysis was performed during 180 s in both the pre-crossing and post-crossing rest periods. White arrows indicate the different Brodmann areas.

According to swLORETA, the alpha oscillation originated from the bilateral parietal cortex (BA7) during the pre-crossing rest condition (Fig. 3, left), while only the superior parietal lobule (BA7) of the left side was identified during the post-crossing rest (Fig. 3, right). This was supplemented by a network that included the right postcentral and marginal gyri (BA40) and the precuneus (BA19). The parietal cortex (BA7) was also identified as a generator of delta and theta oscillation during the pre-crossing rest. Under this condition, the angular (BA39) and supramarginal (BA40) gyri of the right side, and the left inferior parietal gyrus (BA40), were also identified as delta generators; and the middle frontal (BA8) and precentral (BA9) gyri were identified as theta generators. During the pre-crossing rest, beta oscillation was mainly generated by a right-side network formed by the inferior parietal gyrus (BA40), the superior and transverse temporal (BA39) gyri, and the postcentral gyrus (BA3). In contrast, during the post-crossing rest, only the right parietal gyrus was identified as a beta generator (Fig. 3).

### EEG dynamics and generators linked to TA activation

**Figure 4:**
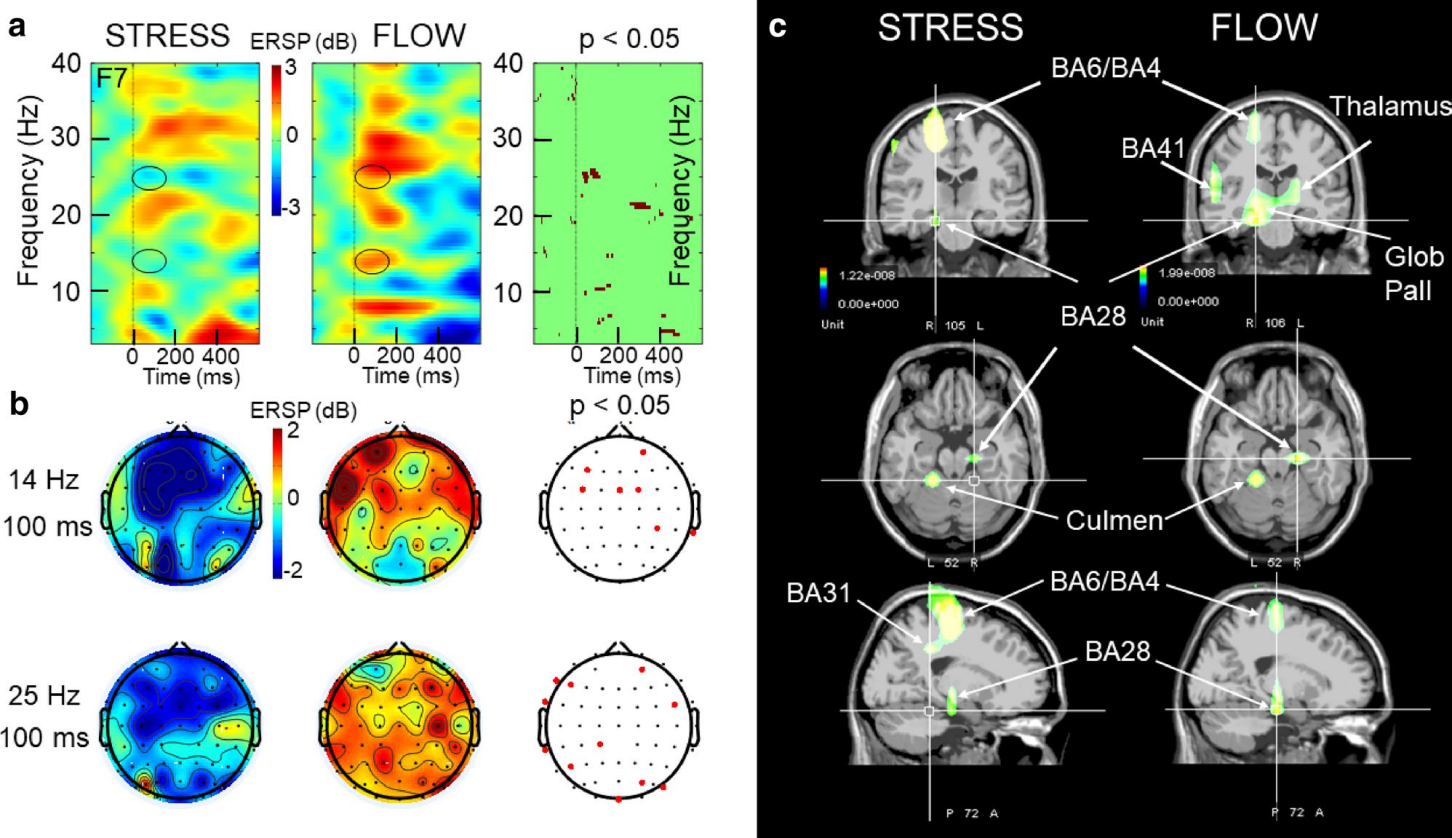
EEG dynamics and neural generators triggered by tibialis anterior (TA) activation. **a** Event-related spectral perturbation and swLORETA related to TA muscle long-burst activation recorded during the STRESS and FLOW. **b** Topography of the low (14-Hz) and fast (25-Hz) beta during STRESS (left) and FLOW (middle) at about 100 ms after TA activation. The third column represents the statistical map (permutation with Holms test *P* < 0.05) corresponding to these beta oscillations. **c** Results of swLORETA identified generators corresponding to EEG activity (beta oscillation) recorded during 1 s after the onset of the long burst of the left TA muscle.

We performed event-related spectral perturbation (ERSP) analysis on the first second of the long-burst activation of the left TA muscle during the FLOW and STRESS (Fig. 4). This revealed significant differences between these two periods. During the first 100 ms after TA activation, we observed a contrasting dynamics of beta oscillation (14–25 Hz) on the fronto-central electrodes—with a power increase [considered an event-related synchronization (ERS)] present during the FLOW, and a power decrease of beta [considered an event-related desynchronization (ERD)] present during the STRESS. Additionally, during TA activation at between 400–500 ms, we observed an alpha (10 Hz) ERS during the FLOW, but not during the STRESS.

Application of swLORETA on these EEG dynamics identified the left cerebellar culmen, left parahippocampus (BA28), and premotor and motor cortex (BA4/BA6) of the right side during both periods. On the other hand, the right globus pallidus, left thalamus, and right BA41 were identified only during the FLOW, and the right BA31 was identified only during the STRESS.

### EEG generators during the performance

**Figure 5:**
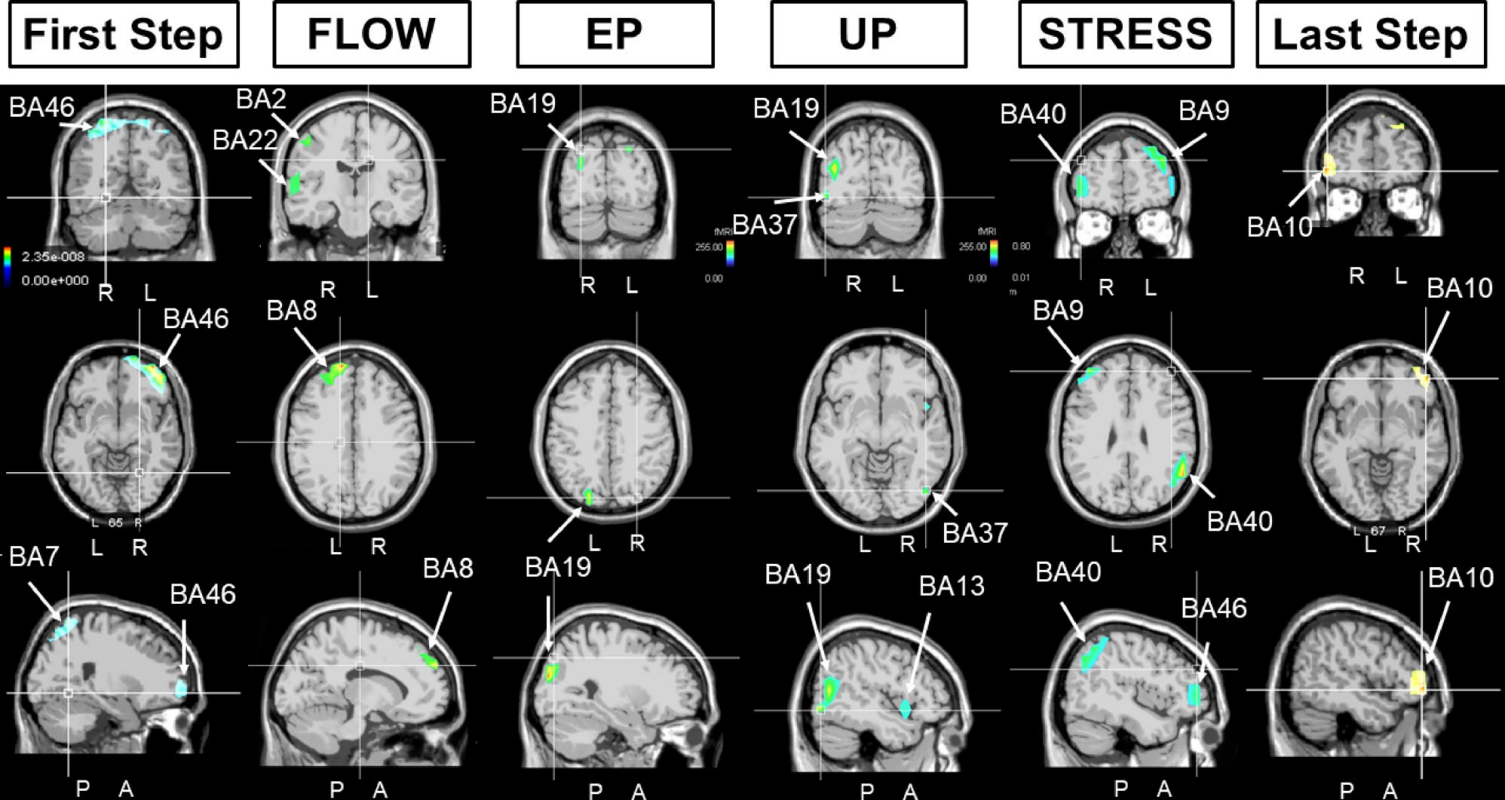
Results of swLORETA during the four main crossing periods (FLOW, EP, UP, and STRESS) for alpha oscillation (7.5–12 Hz). The first and last columns are related to the first and last two periods of the crossing performance.

As in the pre-crossing rest period, during the first two periods of the performance, the alpha generators remained mainly situated in the parietal cortex (BA7) (Fig. 5). However, during crossing, the contribution of BA7 was bilateral and a new area in the right inferior frontal cortex (BA46) was also identified. During FLOW, this network of alpha generators was replaced by another network that included the left superior frontal gyrus (BA8), right postcentral somatosensory cortex (BA2), and right superior temporal gyrus (BA22). During the EP, the main alpha generators were the secondary visual cortex, left cuneus, and right middle occipital cortex (BA19), in accordance with the urge visual demand for changing the snap hook. During the UP, the identified alpha generators include the right middle temporal gyrus (BA37), insula (BA13), and fusiform gyrus (BA19) of the right side. After this unexpected episode, the STRESS was characterized by recruitment of the left superior frontal gyrus (BA9), right supramarginal gyrus of the temporal lobe (BA40), and right inferior frontal gyrus (BA46) as alpha generators (Fig. 5).

The same generator identification process was systematically performed for the delta, theta, and beta oscillations and (Supplementary Figs. 1, 2 and 3, respectively). These results are summarized in Table 1. During all four crossing periods, the frontal BA10 was identified as a delta generator, accompanied by the frontal BA8 (left) and BA46 (right) and the secondary visual BA19, as well as the temporal BA42 during the FLOW, and the left superior frontal gyrus (BA9) and right angular (BA39) gyrus during the STRESS (Supplementary Fig. 1). The identified theta generators included the right frontal BA8-BA10 and right superior temporal (BA42) gyrus during the FLOW; the right temporal (BA22) and parietal (BA40) gyri during the STRESS; and the left frontal BA9 during both EP and UP (Supplementary Fig. 2). The beta generators were identified as occipito-temporal (BA19-BA22) during the FLOW versus fronto-temporal (BA8, BA46-BA42) during the STRESS, and were situated in the temporal gyrus during the EP and UP (BA37/39-BA21 versus BA37/39-BA22) (Supplementary Fig. 3).

**Table 1:** Summary of the Brodmann areas identified by swLORETA during the FLOW, the expected problem (EP), the unexpected problem (UP), and STRESS periods.

Frequency Band	FLOW period	EP period	UP period	STRESS Period
Delta Rythm (1-3 Hz)	BA8 L	BA10 R	BA10 R/L	BA10 R/L
	BA10 L/R		BA47 R	BA9 L
	BA19 R			BA39 R
	BA42 R			
	BA46 R			
Theta Rythm (3,5-7 Hz)	BA8 R	BA9 L	BA9 L	BA22 R
	BA10 R	BA19 L		BA40 R
	BA42 R			
Alpha Rythm (7,5-12 Hz)	BA2 R	BA19 R/L	BA19 R	BA9 L
	BA8 L		BA13 R	BA40 R
	BA22 R		BA37 R	BA46 R
Beta Rythm (15-25 Hz)	BA19 R	BA21 L	BA37 R	BA8 L
	BA22 R	BA37 R	BA39 R	BA42 R
		BA39 R	BA22 R	BA46 L

**Figure 6:**
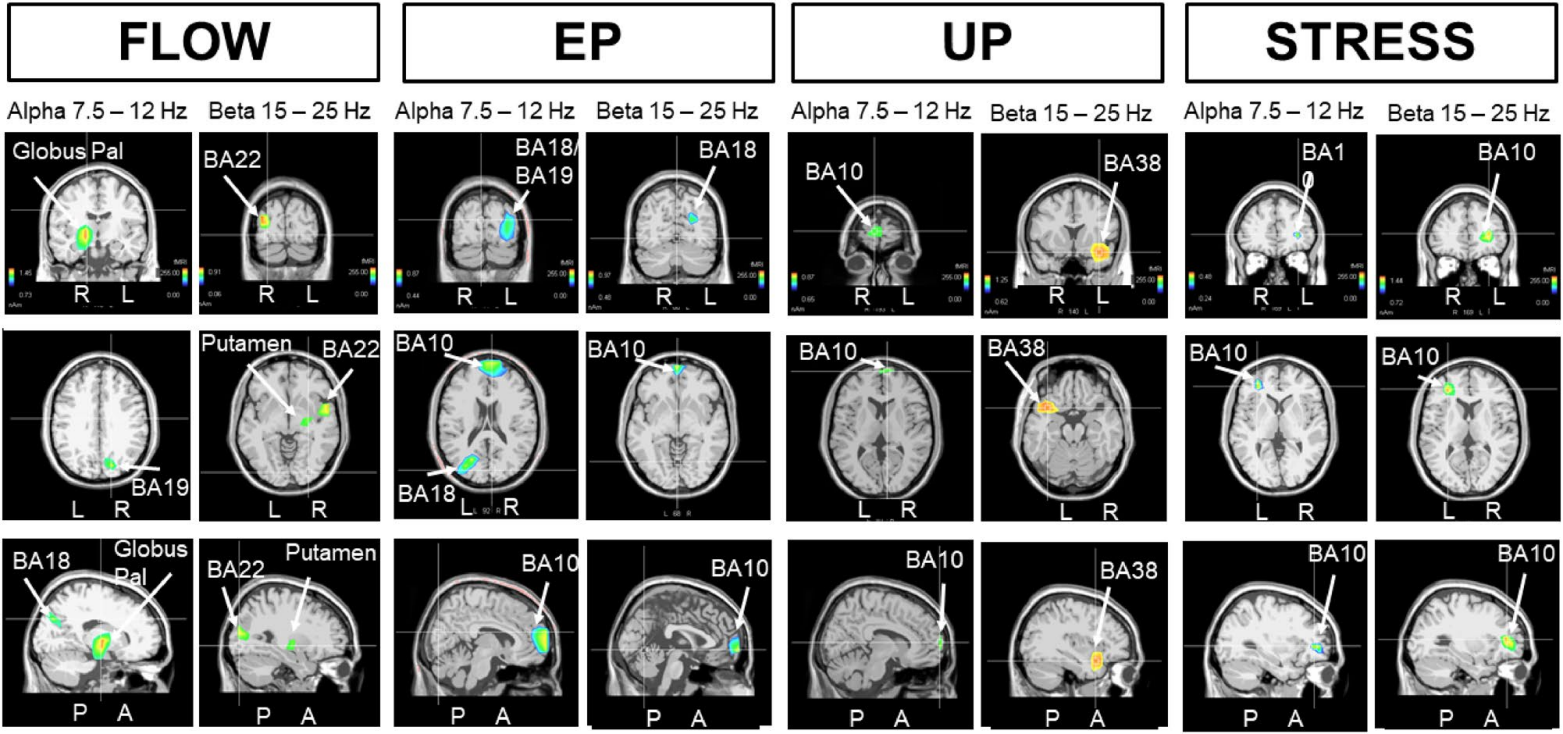
Non-parametric statistical maps of swLORETA-identified EEG generators of alpha and beta oscillations during FLOW versus STRESS, EP versus UP, UP versus EP and STRESS versus FLOW.

To determine which sources were more active during a particular state (e.g., the FLOW) with respect to another state (e.g., the STRESS), we generated nonparametric statistical maps. Figure 6 presents these statistical maps for the alpha and beta oscillations. This analysis unexpectedly revealed increased contributions from the globus pallidus and putamen as generators of alpha and beta oscillations during the FLOW with respect to the STRESS. In the reverse situation (STRESS with respect to FLOW), the statistical comparison indicated an increased single contribution of the frontal BA10. The greater participation of the right globus pallidus and right putamen was accompanied by increased contributions of BA18/19 and BA22 on the same side for the alpha and beta oscillations, respectively.

**Figure 7:**
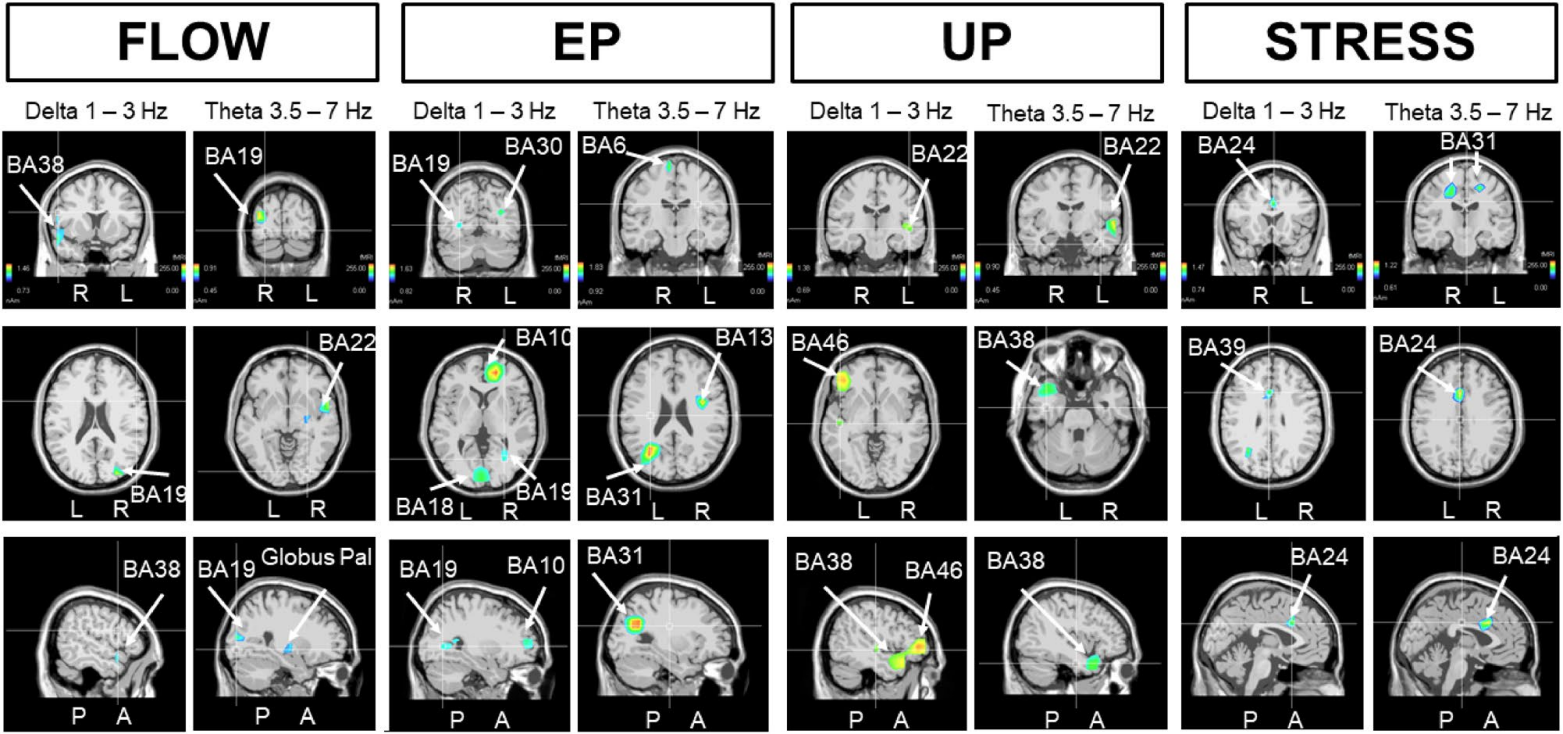
Non-parametric statistical maps of swLORETA-identified EEG generators of delta and theta oscillations during FLOW versus STRESS, EP versus UP, UP versus EP, and STRESS versus FLOW***.***

These results indicated the existence of contrasting EEG sources during a state qualified as “flow” (FLOW) with respect to a more stressed state (STRESS). Interestingly, performance of the same statistical analysis for the theta oscillation also revealed greater participation of the right globus pallidus (Fig. 7), which was accompanied by contributions of the right BA19 and BA22. Importantly, for each analyzed oscillation type, comparison of the “flow”-related FLOW state with respect to the STRESS state never revealed an increased contribution of the frontal cortex (BA10). In contrast, when STRESS was compared to FLOW, the bilateral cingulate cortex (BA31-BA24) showed an increased contribution to the theta oscillation. In this situation, the midline parts of BA24 and BA31 exhibited increased participation towards the delta oscillation. On the other hand, in the comparison of FLOW versus STRESS, we detected stronger contributions of the superior temporal gyrus BA38 and middle occipital gyrus (BA19), both of the right side.

When the same comparative analysis was performed on EP versus UP, or UP versus EP, we never observed any contribution of the subcortical structures (globus pallidus/putamen) to any oscillation type. In these highly complex behavioral situations, different regions of the frontal cortex (BA10) were identified for the alpha contribution during both EP and UP, while the left visual cortex (BA18) was revealed as a stronger contributor during EP compared to during UP. This was also largely the case for the beta oscillation (Fig. 6). However, when comparing the UP to EP, the left superior temporal region (BA38) was a stronger contributor to beta oscillation. Interestingly, BA38 was also identified when the same comparative analysis was applied to the delta and theta oscillations (Fig. 7). The temporal BA22 and frontal BA46 were also stronger for the delta oscillation, and only BA22 was stronger for the theta oscillation. Comparison of the EP versus UP revealed different delta and theta oscillation sources than the reverse comparison (Fig. 7). For the delta oscillations, the stronger source contributors included the occipital (BA19), frontal (BA10), and posterior cingulate (BA30) cortex—as already observed (BA10 and BA19) for alpha oscillation (Fig. 6). For theta oscillations, the left BA31, left BA6, and right BA13 were recruited when the action was expected (EP) rather than unexpected (UP).

## Discussion

The high skill level of a professional performer does not preclude the possibility that unpredictable challenging events will occur—for example, the block of the snap hook that occurred during our recorded tightrope performance. This disturbance in our presently analyzed recording enabled the unique possibility of comparing the emergence of the flow state early in the performance (FLOW) with the more stressed period that occurred during the last period of the crossing (STRESS). This single recording of a professional tightrope performer is the first demonstration that it is possible to record the EEG pattern during this unique balance exercise under ecological condition.

The PRSA profile discriminated a small peak of alpha oscillation only during the FLOW, possibly indicating that alpha oscillation was present during this flow state and less prominent during the STRESS. Since the STRESS occurred during the last part of the crossing performance and after the UP, we must consider the possible influence of fatigue. However, the influence of mental and physical fatigues on EEG dynamics has not yet been resolved, and appears to largely depend on the behavioral paradigmatic context^31^. For example, attentional cognitive demands are reflected by a decreased alpha oscillation and increased theta in the parieto-occipital and frontal regions, respectively^32^. However, fatigue related to driving performance and inducing driving error^18^ and fatigue-related increased reaction time are reportedly accompanied by an increased alpha power oscillation^31^, and not the decreased alpha power that was reported in the present situation.

Here we compared the EEG generators obtained by the 3D inverse solution method (swLORETA) during different periods delimitated by the occurrence of an unpredictable problem. This enabled the identification of significant cortical and subcortical EEG generators that corresponded to different brain states: one period close to the ‘flow’ (FLOW) and another more closely related to a stress situation (STRESS). While the sensory-motor demand remained largely constant throughout the crossing performance, the present study is the first to characterize a rapid change in EEG generators, influenced by external predicted and unexpected events that substantially altered the performer’s mental set. Tightrope performance requires rapid access to the subcortical and cortical networks sustaining the sensory-motor demands (motor planning and coordination), with cognitive and emotional factors supporting performance optimization. The observed rapid modification of the EEG generators’ localization in cortical and subcortical areas corroborates the previously reported rapid dynamics of MEG dipoles^33,34^. Moreover, the rapid changes in cortical or subcortical areas during the tightrope performance may inform the effective dynamics between cognitive-emotional states, ranging from negative stress to flow, and the control of motor equilibrium challenges.

Among the different forms of balance exercises and control-requiring locomotion^35,36^, tightrope performance is one of the most dynamic tasks, requiring postural coordination from head to trunk, and foot placement during the step-to-step progression along the cable^37,38^. Moreover, this difficult task is facilitated by the use of a balancing artificial arm, which must be considered in the interpretation of our present results. Overall, the EEG signal dynamics may be considered as the integrated mixing of different outputs (planning and executive commands) and inputs (feedback signals), which are also present in a simple form during human bipedal locomotion^39–41^.

We identified a theta peak during the most difficult balance period of the tightrope crossing. This corroborates a 2016 report by Hülsdünker et al.^42^, showing increased theta power in the frontal, central, and parietal regions when a balance task became more challenging. This finding is also compatible with evidence that the vestibular system can modulate theta oscillation^43^.

The majority of the identified EEG generators in our study were very similar to those described by la Fougère et al. in 2010, based on PET and fMRI during real and imagined locomotion. La Fougère et al.^44^ identified 22 BA as activation areas during locomotion execution and planning, of which 12 were identified by swLORETA in our present study. Moreover, of the 58 BA identified during the six periods in the present study, 33 BA were also previously identified using PET. Finally of the 13 BA highlighted in the comparative analyses between FLOW versus STRESS, EP versus UP, and vice-versa, only 6 BA (BA2, BA8, BA28, BA38, BA42, and BA46) were not also identified during locomotion by la Fougère et al.^44^. Thus, despite the differences between simple locomotion and tightrope performance, the presently identified EEG sources seem to be robust and in accordance with the findings of PET and fMRI locomotion studies. Among the six areas identified only in tightrope walking, the somatosensory BA2 is involved in multimodal sensory imagery^45,46^, BA8 reflects decision-making processes reported in a simple movement (key press task in response to visual task)^47^ that is very different from the present sensory stimulation and motor task. In addition, BA28 is implicated in action observation^48^, BA38 in emotion and attention^45,46^, BA42 in threat representation^49^, and BA46 in the resolution of attentional-perceptual conflicts^50^. These six BA are particularly interesting because they are likely not directly linked to real or imagined walking activities, as the areas reported by La Fougère et al.^44^. We thus speculated that they could be linked to tightrope specificities or to the related FLOW or STRESS states.

Our present analysis of the FLOW (flow state) with respect to the STRESS (stress state) demonstrated that the right superior temporal gyrus (BA38) and right globus pallidus and putamen were generators of the delta, alpha, and beta oscillations, respectively. In contrast, in the STRESS with respect to the FLOW, we identified the middle temporal gyrus (BA39) as the generator of delta oscillation. The medial frontal gyrus (BA10) is a frontal area involved in inhibitory control^51^ and has been previously identified in walking imagery^44^. In our present study, BA10 was identified in the STRESS with respect to FLOW but not in the FLOW with respect to STRESS analysis. These findings corroborate the theoretical proposal of Dietrich^15,16^ that flow state emergence requires transient hypo-frontality to momentarily suppress the analytical meta-conscious abilities of the explicit system, in favor of the implicit system mainly localized in the basal ganglia circuit^53,54^.

The left superior temporal gyrus (STG, BA38) is dedicated to language processing, while the right side is considered the center of spatial awareness^55^. It is also important for social emotion processing^56^ and facial emotion^57^. Interestingly, adolescents with a suicide attempt history show reduced grey-matter volume in the right BA38^58–60^. The right STG is also implicated in gaze processing, as demonstrated in the rare case of a completely lesioned BA38^61^. Despite the potential contribution of BA38 in complex visual treatment (exploratory visual search task)^62,63^ and gaze control^61^, the crossing task remained the same during the FLOW and the STRESS, and during the snap hook problem (EP-UP). This may indicate that the gaze control functions of the right BA38 are not the reason for the emergence of this area only when analyzing the FLOW compared to STRESS, and the UP compared to EP. It may be more likely that the right BA38 emerged upon encountering the main characteristics of the flow state (balance between challenges and skills). Following this line of reasoning, the right BA38 (delta generator) may be considered responsible for the emotional content (overcoming the fear of falling) of the “flow”, which could result from the integration of multiple executive functions into an egocentric spatial awareness network including the right BA38^64^.

Interestingly, our present identification of basal ganglia (putamen and globus pallidus) in conjunction with BA38 is in accordance with anatomical^65,66^ and clinical^55^ data demonstrating extensive connections between the right STG (BA38) and the basal ganglia. Notably, lesions of these cortico-subcortical structures (including the thalamic pulvinar) cause spatial neglect. In contrast, the BA39 identified in the comparison of STRESS to FLOW is not crucial for spatial awareness, but rather involved in longer-term coding for reaching control of targets^67,68^. This function could be useful during the STRESS, when the performer reported that he remained anxious about the possible locking of his carabiner during the last part of the tightrope performance. We identified the left middle frontal gyrus (MFG; BA10) as a generator of alpha and beta oscillation during STRESS, and the right + left MFG during UP and EP, which contrasts with its absence during FLOW. TMS has revealed that the left MFG is related to selective visual attention tasks, while the right MFG is more implicated in sustained attention^69^. Functional MRI has also identified the MFG as an important node of the inhibitory control network, which comprises 25 connections involving nodes in the orbitofrontal cortex, the inferior frontal gyrus, and the limbic, occipito-parietal areas^51^.

The present real ecological situation did not enable the measurement of tightrope kinematics. However, the recording of the bilateral TA EMG muscles showed a pattern of long and short burst sequence alternation indicating that the analyzed crossing was consistently performed, except during the snap hook problem. Due to this EMG pattern conservation of one of the main walking elements for the step-to-step progression across the cable, we may assume that the differences in EEG generators during FLOW and STRESS were not due to gross kinematic differences in the movement performance, but rather to a drastic change in the more general brain state related to flow or negative stress.

In this study, we used two main inverse modelling analyses (swLORETA)—one based on self-reported mental states, and the other based on the motor commands. These analyses were complementary and enabled identification of the EEG generators involved in the motor control of a tightrope performance, and in the different mental state periods related to this performance. The EEG generators identified during the TA burst were in accordance with an on-line control exerted by the ipsilateral cerebellum and the contralateral sensorimotor cortex during both the FLOW and the STRESS. These well-known cerebello-cortical pathways reinforce the physiological plausibility of the present identifications made using a mobile EEG system during extreme sport performance. Additionally, we identified the right globus pallidus when EEG periods were triggered by TA activity onset only during the FLOW, which reinforces the same identification in comparisons of FLOW relative to STRESS, regardless of the TA sequences. Overall, these findings support the notion that the flow state is accompanied by recruitment of additional EEG generators (globus pallidus) that differ from those already linked to the on-line control of the skill movement (cerebellum and parahippocampus).

## Methods

### General condition

Informed consent was obtained from the subject. The ethics committee of the Université Libre de Bruxelles has approved all methods carried out in the study. All protocols in the study are in accordance with relevant guidelines and regulations and have been conducted in conformity with the European Union directive 2001/20/EC of the European Parliament. The subject is a professional and he accepted to participate in this unique experience during one training or repetition session 2 days before the definitive prestation in the presence of public. In order to avoid psychological interference with his training habits, the performer realized this performance at his proper tempo without any specific instructions from the experimenter. He reported that in such conditions, he may encounter the flow sensation as the one experienced in the presence of the public. (A video illustrates the performance). The psychometric measure of the flow was assessed by the Flow Short Scale questionnaire and a video illustrates the performance (see Supplementary information).

### EEG, EMG, and EKG recordings

During the unique tightrope performance, we continuously monitored EEG activity using an elastic shielded cap containing 64 scalp electrodes (Eegosports system of ANT). The sampling frequency was 512 Hz and the resolution was 22 bits (71.5 mV per bit). EEG data were recorded on a computer tablet placed on the subject’s back. All electrodes were referred to the CPz electrode. We also recorded two electro-oculograms (for horizontal EOG signals), two electromyograms for detecting any activity related to real movement [tibial anterior (TA) right and left], and two electrocardiograms using a bipolar EKG lead II configuration. Off-line data treatment and statistical analyses were performed using EEGLAB software^70^, ASA software (ANT neuro system), and in-house MATLAB-based tools.

### Description analysis method

Based on video observation and the performer’s report, we divided the EEG data into six periods: two rest periods (pre-crossing and post-crossing) and four periods during the crossing. The crossing was divided into the period before the expected changing of the lifeline, when the performer reported being in the flow (quiet crossing, FLOW); the period when he changes his carabiner (expected problem, EP); the period when the carabiner is blocked (unexpected problem, UP); and when the performer reported remaining stressed (mindful crossing period, STRESS). These divisions were confirmed by EKG analyses (Fig. 1).

### EEG analysis

We initially applied a 45-Hz low-pass filter and a 0.5-Hz high-pass filter. Then any artefactual portions of the EEG data were rejected by visual inspection. Next, independent component analysis (ICA) was performed on continuous data to detect and reject synchronous or partially synchronous artefactual activities (mostly blinks).

### Phase-rectified signal averaging

The cleaned EEG signals were further analyzed using a phase-rectified signal averaging (PRSA) method^71^. The PRSA algorithm is used to study quasi-periodic oscillations in nonstationary and noisy signals, and has been successfully applied in EEG studies^72^. PRSA was applied to the two rest periods, and to the four selected crossing periods (Fig. 2e, f).

### Event-related spectral perturbation

The EEGLAB software enables analysis of event-related dynamics, and deciphering of ongoing EEG processes that may be partially time-locked and phase-locked to experimental events^73^. The ERSP may correspond to a narrow band of event-related desynchronization (ERD) or synchronization (ERS). Briefly, for this calculation, EEGLAB computed the power spectrum over a sliding latency window in each trial, and then the average across trials. Following the gain model^70^, for each frequency band at each frequency point, the power spectrum was divided by the averaged spectral power during the pre-stimulus baseline period (− 0.5 s to 0 s). Log-transformation of this measure allowed the visualization of a wider range of variation^74^.

Each trial included samples from 1 s before and 2 s after the stimulus. Each image pixel was color coded to indicate the achieved power (in dB) at a given frequency, and the latency relative to stimulation onset. For n trials, if *F*_*k*_*(f, t)* was the spectral estimate of trial *k* at frequency *f* and time *t*:

1 $${\text{ERSP}}\left(f,t\right)=\frac{1}{n}\sum _{k-1}^{n}{\left|{F}_{k}\left(f,t\right)\right|}^{2}$$

To compute *F*_*k*_(*f*, *t*), we used the short-time Fourier transform function of the EEGLAB software, providing a specified time and frequency resolution. To quantify ERSP in terms of time and frequency, we calculated the ERSP volume expressed in arbitrary units. To increase the time resolution, we used wavelet transform to measure the onset and termination times of the ERS and ERD periods. Wavelet transform was applied for complex spectro-temporal representation with Hanning-windowed sinusoidal wavelets at 1 cycle (lowest) to 12.5 cycles (highest). ERSP templates were calculated with 200 time-points, from − 721.5 ms to 1,221.5 ms, using a window size of 285 samples (556.6 ms) at 97 linear-spaced frequencies from 2 to 50 Hz. For the significance level of ERSP, we used bootstrap resampling (*P* < 0.05) as a surrogate method. Under this condition, temporal accuracy (∆t) was calculated using the following formula: ($\Delta t = \frac{1/2\pi }{{\Delta f}}$), yielding a result of 16.3 ms.

With baseline correction (− 0.5 s to 0 s), the averaged spectral power was extracted from − 0.5 s before to 2 s after the start of the long burst of the left anterior tibial muscle. We used a total of 49 epochs for the FLOW period, and 50 epochs for the STRESS period.

#### Inverse modeling method (swLORETA)

For source reconstruction, we used standardized weighted low-resolution electromagnetic tomography (swLORETA)^25^, which is a distributed inverse solution method that can model spatially distinct sources of EEG signals without a priori knowledge about the generator’s location. Derived from the sLORETA method^74^, swLORETA has enabled accurate reconstruction of surface and deep current sources, even in the presence of noise and with two simultaneously active dipoles. This is achieved by incorporating singular value decomposition-based lead field weighting, which compensates for the varying sensitivity of the sensors to current sources at different depths (Palmero-Soler et al. 2007).

In LORETA analysis, the data were re-referenced to the average reference. We used a realistic boundary element model (BEM) to solve the forward problem with a probabilistic brain model^74^. The solution was computed using 2030 voxels (5.00-mm grid spacing), and restricted to the gray matter of the cerebrum and cerebellum based on tissue maps available from the Montreal Neurological Institute^74,75^. We obtained Talairach coordinates for every voxel by placing the corresponding Talairach markers onto the anatomical template^76^. The corresponding brain areas were labeled based on the final coordinates of the maxima values (x, y, z) from the Talairach atlas coordinates. Cerebellar regions were defined using the nomenclature of the MRI Atlas of the Human Cerebellum of Schmahmann^77^.

#### EEG source analysis

To identify the EEG generators (beta oscillation) corresponding to the long burst of the TA muscle, we focused on the averaged spectral power of 49 epochs during the FLOW period and 50 epochs during the STRESS period, extracted from − 0.5 s before to 2 s after the start of the long burst of the left TA muscle. For generator identification, we selected the time-point of 1 s from TA activation.

We also analyzed EEG signals unrelated to muscle activation, but focused on the general mental state during different periods of the EEG recordings. For source reconstruction, we performed visual observation to determine the successive slicing of 13 boots of 2-s duration for each of the four crossing periods. We then identified the EEG sources of the delta, theta, alpha, and beta rhythm. This allowed us to perform statistical comparisons between the identified sources corresponding to these four behavioral periods that were already validated by both behavioral events and heartrate modifications along the crossing.

### Statistical analysis

For statistical analysis between physiological parameters (∆t and RR), we performed one-way ANOVA testing using Statistica 7.0 software. The results were expressed as mean ± SD, and a *P* value of < 0.05 was considered significant. To examine significance of the ERSP from the full scalp array, we employed a parametric method provided by the EEGLAB software^70^. We examined the general mental state using a nonparametric permutation test^78^ on the 13 selections of 2-s duration, with a *P* value of < 0.05) indicating statistical significance of the current density magnitude. We also used the paired t-test for swLORETA solutions to compare the different periods throughout the crossing—[FLOW-STRESS], [STRESS-FLOW], [EP-UP], and [UP-EP]—with the null hypothesis that there was no difference between the compared conditions. We used the 95th percentile of the calculated permutation distribution for the maximal statistics, defining a 0.05 level of corrected significance threshold. In other words, we could reject the null hypothesis for any voxel with a t-value of the un-permuted T image greater than the 95th percentile of the permutation distribution of the maximal statistics^78^.

## Supplementary information

Supplementary file1 (DOCX 2599 kb)

Supplementary file2 (MP4 61564 kb)
