# Supplementary material for: EEG dynamics and neural generators of psychological flow during one tightrope performance
Source: Sci Rep. 2020 Jul 24;10:12449. doi: 10.1038/s41598-020-69448-3 (PMC7381607; doi:10.1038/s41598-020-69448-3)
Supplement: Supplementary file 1 — Supplementary file1 (DOCX 2599 kb) [file 41598_2020_69448_MOESM1_ESM.docx]

**Supplementary Information**

**EEG dynamics and neural generators of psychological flow during one tightrope performance**

**A. Leroy^1,3^ and G. Cheron^1,2^**

*1.Laboratory of Neurophysiology and Movement Biomechanics, Université Libre de Bruxelles, Brussels, Belgium,*

*2. Laboratory of Electrophysiology, Université de Mons-Hainaut, Mons, Belgium*

*3. Haute Ecole Prinvinciale du Hainaut-Condorcet*

**Supplementary materials**


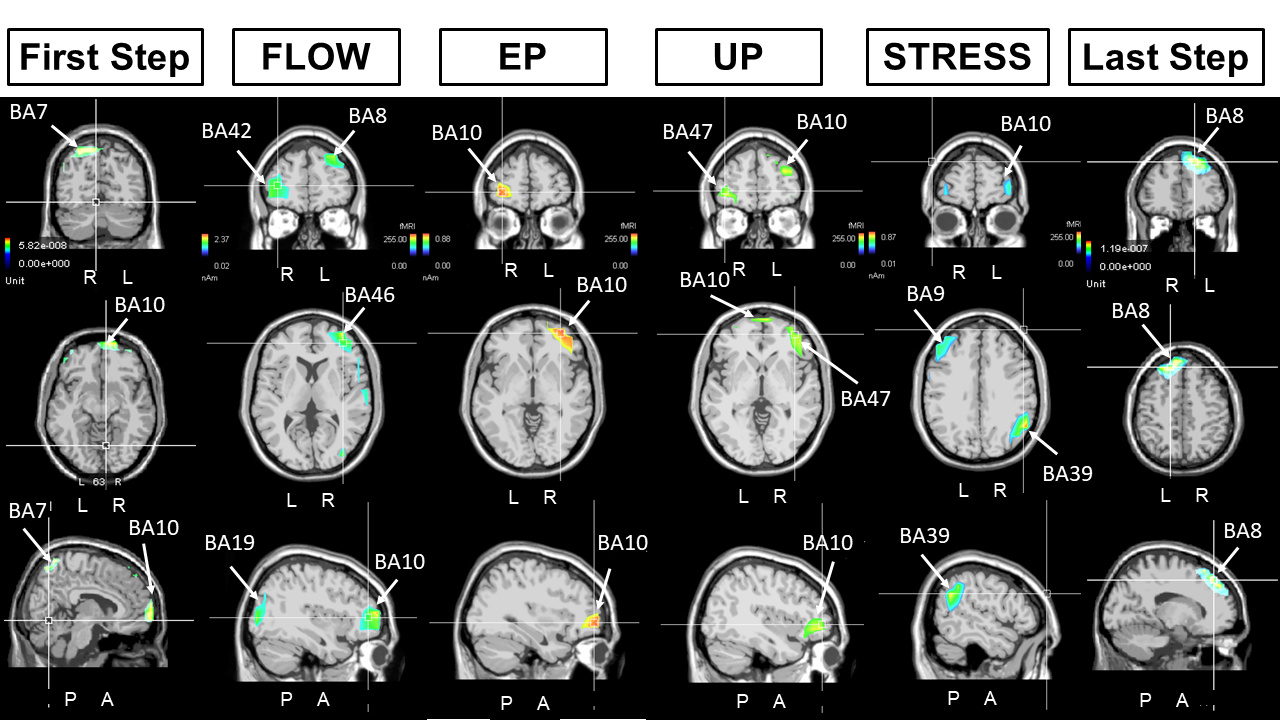


**Supplementary Figure . 1:** Results of swLORETA during the four main crossing periods (FLOW, EP, UP, and STRESS) for delta oscillation (7.5–12 Hz). The first and last columns are related to the first and last two periods of the crossing performance.


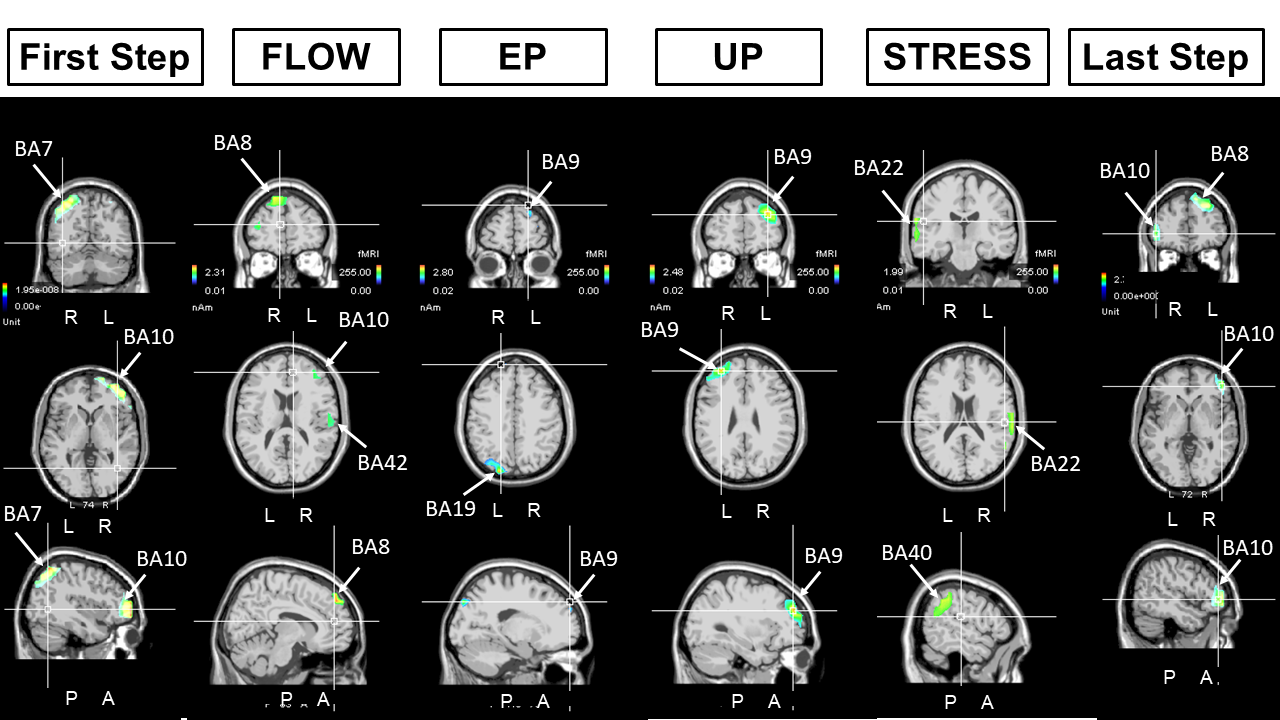


**Supplementary Figure 2**:. Results of swLORETA during the four main crossing periods (FLOW, EP, UP, and STRESS) for theta oscillation (1–3 Hz). The first and last columns are related to the first and last two periods of the crossing performance.


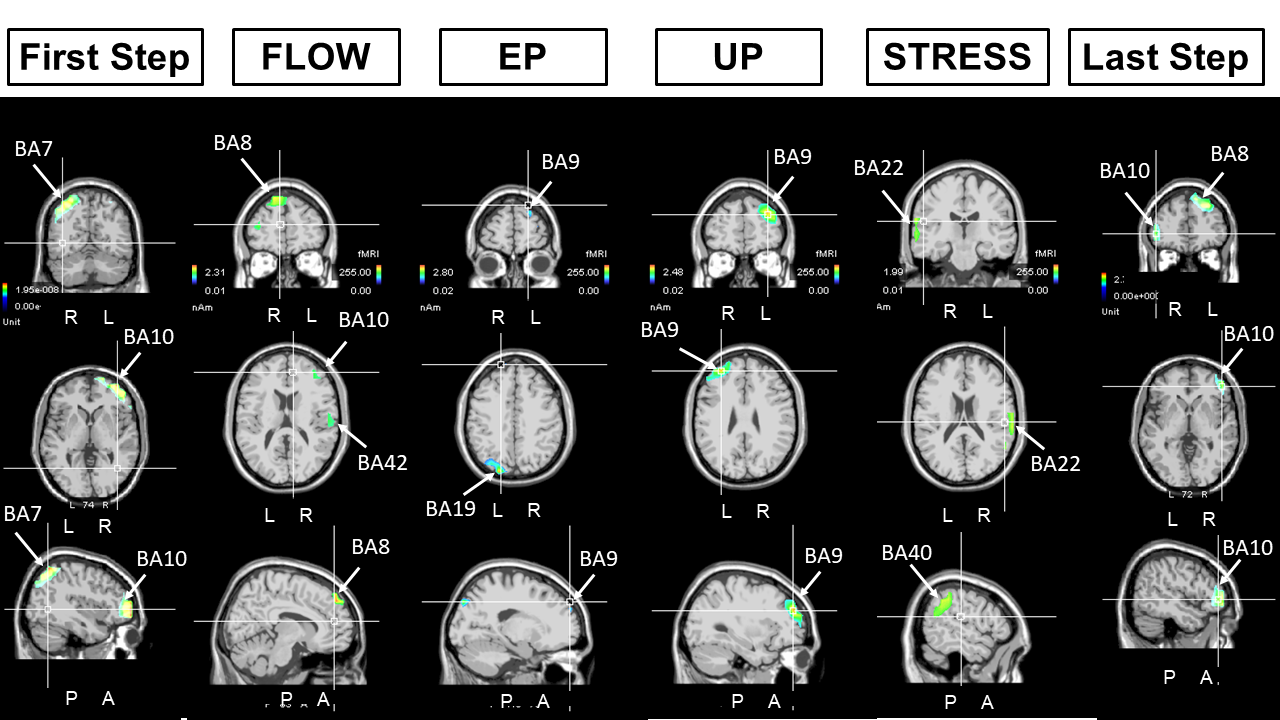


**Supplementary Figure 3**: Results of swLORETA during the four main crossing periods (FLOW, EP, UP, and STRESS) for beta oscillation (15–25 Hz). The first and last columns are related to the first and last two periods of the crossing performance.

**Verbal reports of flow sensation**

- “I am in harmony with my feelings, with my body, with the achievement that it is performing; each of my gestures becomes fluid, I feel good ; I fully sense my resources. There, for example, above the trees, with a small green sea below, it's really a moment when I think it's the ‘flow’, a moment of grace when I feel good, when things are going well!” (*Oliver Zimmerman, the present performer*).
- “It was a good performance, my heart is still beating fairly hard, I felt internal heat ! In fact, it’s a state that allows me to feel good and to be able to tell something deeper” (*Magali Richette, guitarist*).
- “The ‘flow’ is when you’re on a cloud, you know that every shot will be OK, that’s what any athlete is looking for and so you’re constantly trying to replicate it. In fact, ou're working on it, trying to get back into that state of 'flow' “ (*Evan Fournier, basketball player*).
- “When you arrive in the flow, you call that "the artist", you fly! You don't have many moments like that in your career, normally you feel you are in control of everything, including of your opponents. When you get there, you tell them ‘disappear ’! It doesn't matter ‘I’m coming’” (*Ladji Doucouré, sprinter*).
- “I had a pretty creative universe when I was a little girl and I have the feeling that when I sing and come close to the ‘flow’, it’s a moment when I can find that feeling again and be completely free. I feel a kind of total well-being, I am in tune with myself”. (*Valentine Brugnion, singer*).

**Flow Short Scale questionnaire**


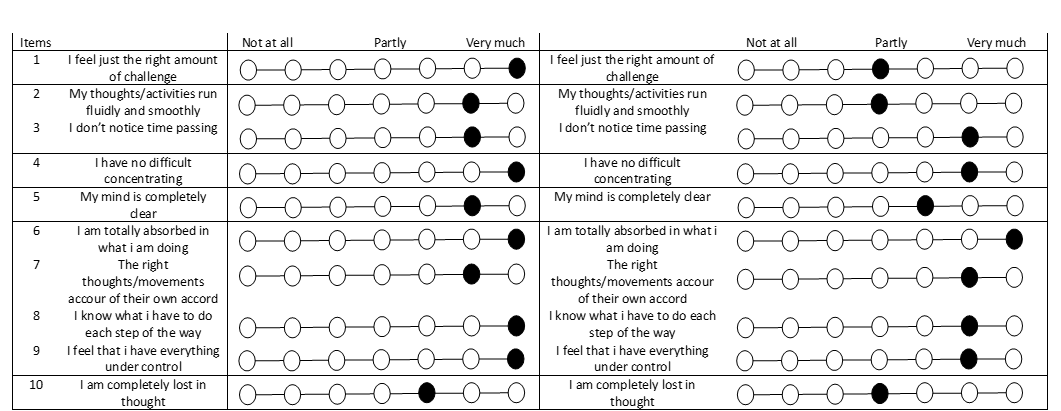


Flow-experience was also assessed by Flow Short Scale (FSS), (Engeser and Rheinberg, 2008) which was here performed for the two behavioral periods: (1) before the carabiner problem – Flow period (left side of the questionnaire) and (2) after the carabiner problem – Stress period (right side of the questionnaire). The FSS scale contains ten items. The items 1, 3, 6 and 10 evaluated the absorption by activity, while the items 2, 4, 5, 7, 8 and 9 evaluated the fluency of performance. Each of these items are evaluated on a seven-point Likert scale (with 1 = “ I don’t agree” and 7 =” I agree”). Although that the psychometric measure of the flow is complex and not robust, the internal consistency of the FSS has been evaluated by a Cronbach’s alpha of 0.77 and 0.88 for the subscale absorption and subscale fluency, respectively. The mean FSS score was of 6.4 ± 0.69 for the Flow and 5.4 ± 1.07 for the Stress period. This difference was statistically different with a p-value = 0.017 at the paired Wilcoxon test showing that the two periods were psychometrically distinct.

**Reference**

Engeser, S. and Rheinberg, F. Flow, performance and moderators of challenge-skill balance Motiv Emot (2008) 32:158–172. DOI 10.1007/s11031-008-9102-4

**
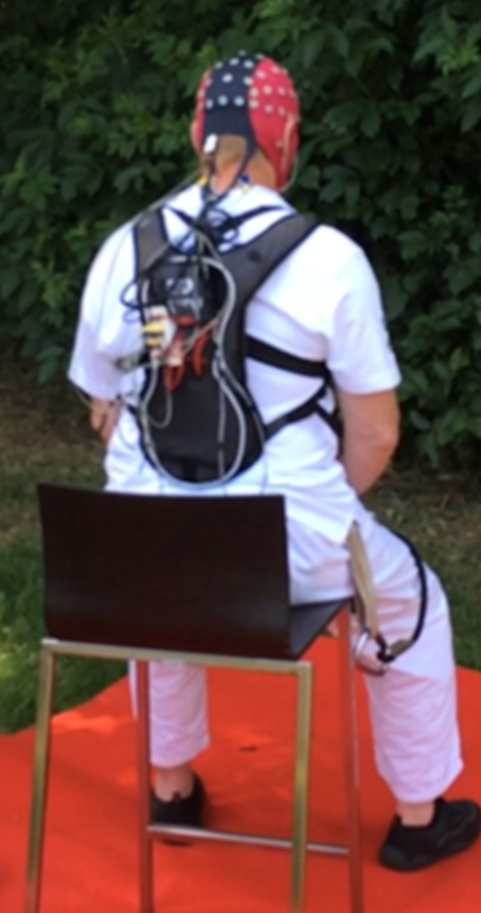
**

**Supplementary Figure 4. Illustration of the portable device.** The EEG activity recorded using an elastic shielded cap containing 64 scalp electrodes (Eegosports system of ANT) were recorded on a computer tablet placed on the subject’s backpack specifically designed for such sport performance.


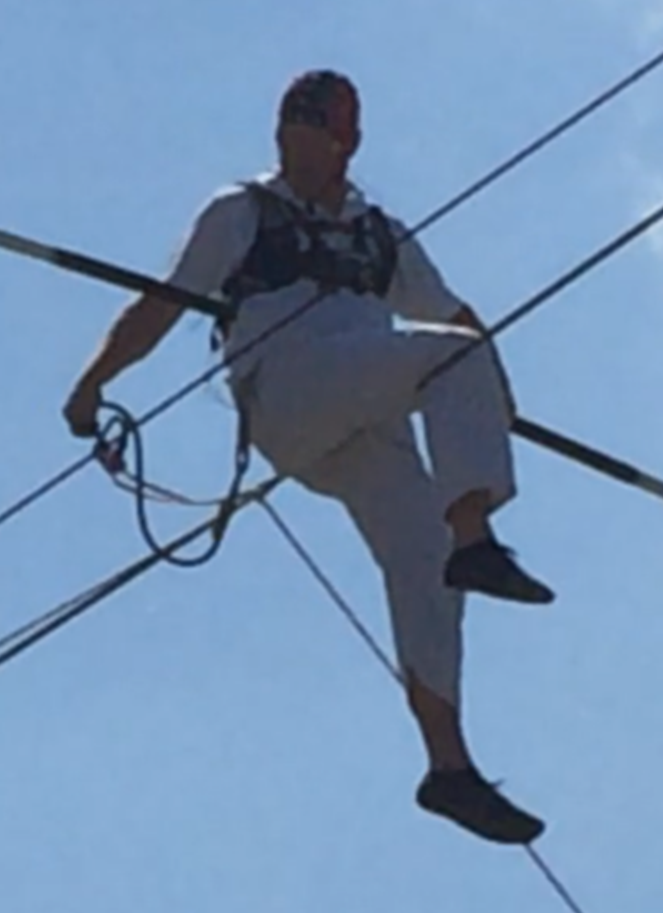


**Supplementary Figure 5**. Illustration of the changing of the carabiner
